# Evaluation of pharmacokinetics of warfarin from validated pharmacokinetic-pharmacodynamic model

**DOI:** 10.5599/admet.909

**Published:** 2021-01-18

**Authors:** Kannan Sridharan, Rashed Al Banna, Aysha Husain

**Affiliations:** 1 Department of Pharmacology & Therapeutics, College of Medicine & Medical Sciences, Arabian Gulf University, Manama, Kingdom of Bahrain; 2 Department of Cardiology, Salmaniya Medical Hospital, Ministry of Health, Manama, Kingdom of Bahrain; 3 RCSI-MUB, Manama, Kingdom of Bahrain Full Affiliation, Address

**Keywords:** Pharmacokinetics, Warfarin, Anticoagulant

## Abstract

**Background:**

Pharmacokinetics of warfarin has not been described in our population. We derived the pharmacokinetic parameters from a validated pharmacokinetic-pharmacodynamic model.

**Methods:**

Patients receiving warfarin for at least 6 months were recruited and their demographic characteristics, prothrombin time international normalized ratio (PT-INR), warfarin doses and concomitant drugs were collected. Using a validated pharmacokinetic-pharmacodynamic model, we predicted maximum plasma concentration (*C*_max_), total clearance (*C*_L_), volume of distribution (*V*_d_) and elimination rate (*k*). Warfarin sensitive index (WSI) and warfarin composite measures (WCM) were estimated from the dose and INR values. Liver weight was predicted using validated formula.

**Results:**

Two-hundred and twenty patients were recruited. The following were the predicted pharmacokinetic parameters: *C*_max_ (mg/L) was 5.8 (0.4); *k* (L/day) was 1 (0.1); CL (L/day) was 2.1 (0.2); and *V*_d_ (L) was 7.6 (0.2). Patients with *C*_max_ and elimination rate outside the mean+1.96 SD had significantly lower WSI and higher WCM. Significant correlations were observed between *C*_max_ with CL, *V*_d_, and *k* of warfarin. Significant correlations were also observed between CL and *V*_d_ of warfarin with liver weight of the study participants.

**Conclusion:**

We predicted pharmacokinetic parameters of warfarin from the validated pharmacokinetic-pharmacodynamic model in our population. More studies are needed exploring the relationship between various pharmacodynamic indices of warfarin and pharmacokinetic parameters of warfarin.

## Introduction

Warfarin, an oral anticoagulant, exhibits narrow therapeutic window and poses clinical challenges in maintaining appropriate therapeutic effect. Warfarin is metabolized in liver and its anticoagulant effect is considered optimum when the prothrombin time international normalized ratio (PT-INR) is to be maintained between 2.5 and 3.5 for those undergoing heart valve replacements and between 2 and 3 for others [1]. Warfarin is completely absorbed with time to maximum concentration of 2-6 hours; volume of distribution (*V*_d_) of 10 L; clearance (CL) of 0.2 L/hour; and elimination half-life of 35 hours [2]. Warfarin is a racemic mixture of *S-* and *R-warfarin* posing challenges in measurements [3]. Pharmacokinetic-pharmacodynamic (PK-PD) relationship of warfarin has been well elucidated and therapeutic drug monitoring is carried out by PT-INR measurement [4]. Warfarin pharmacokinetics has not been described in our population. Hence, we carried out the present study to predict the pharmacokinetic parameters of warfarin from the pharmacodynamic variables using a validated PD-PD model.

## Experimental

### Study ethics and design

The study was retrospective carried out as a part of warfarin pharmacogenomics study after obtaining approval from institutional ethics committee and consent from study participants. The present study complies with the latest World Medical Association Declaration of Helsinki guidelines.

### Study procedure

Participants receiving warfarin for at least 6 months were recruited in the present study. Their demographics, PT-INR values, dose and frequency of warfarin, and concomitant drugs were obtained. We assessed CHA_2_DS_2_-VASc, HASBLED and SAMe-TT2R2 scores for the study participants [5,6]. Drugs/drug classes such as statins, proton pump inhibitors, carbamazepine and amiodarone were categorized as drugs with potential interaction with warfarin [7]. We followed National Institute for Health and Care Excellence (NICE) guidelines for classifying anticoagulation control into good (TTR ≥ 65 %) and poor (TTR < 65 %) [8]. We estimated Warfarin sensitive index (WSI) based on the ratio of PT-INR upon the last dose of warfarin [9]. We assessed the difference in PT-INR between the two consequent visits that was squared and divided by the time interval in days. This cross product with the average of PT-INR on the visits followed by square root of this product provided the INR variability. We log-transformed it to base 10 to obtain log-INR variability [10]. Standardized Z scores were obtained for TTR and log-INR variability and warfarin composite measure (WCM) was estimated [11]. Liver weight was estimated by using the validated formula: liver weight (g) = 218 + body weight (kg) * 12.3 + gender * 51 (Male-1; Female-0) [12].

### Predicted pharmacokinetic parameters

We used JPKD software^©^ for estimating pharmacokinetic parameters of warfarin from the validated warfarin PD-PD inbuilt model [13]. The following equations were input in the model:

*C*_s_ = ((1/(-(m*Cl/*V*)/(*k*^2)*(1-(k*Tau/24)/(1-exp(-k*Tau/24)))-(*m*/*k*)*ln((Dose/*V*)/(*C*_max_*(1-exp(-(Cl/*V*)*(Tau/24))))))+3.36)/4.368)^(1/0.383)

INR = ((1/(-(m*Cl/*V*)/(*k*^2)*(1-(k*Tau/24)/(1-exp(-k*Tau/24)))-(*m*/*k*)*ln((Dose/*V*)/(*C*_max_*(1-exp(-(Cl/*V*)*(Tau/24))))))+3.36)/4.368)^(1/0.383)

Dose = exp((1+((m*Cl/*V*)/*k*^2)*(1-(k*Tau/24)/(1-exp(-k*Tau/24)))*(4.368*INR^(0.383)-3.36))/((-*m*/*k*)*(4.368*INR^(0.383)-3.36)))* *C*_max_ *(1-exp((-Cl/*V*)*Tau/24))*V

### Statistical analysis

We represented the demographic details using descriptive statistics. Chi-square test for association was used for assessing the categorical variables. Numerical variables following the assessment of their distributions were tested using Mann-Whitney U test. Correlations between the variables were tested using Pearson correlation tests. A p-value of < 0.05 was considered significant. SPSS version 26 (IBM Corp. Released 2019. IBM SPSS Statistics for Windows, Version 26.0. Armonk, NY: IBM Corp.) was used for statistical analysis. Normal distributions were created for each of the key pharmacokinetic parameters and characteristics were compared between those with values within mean+1.96 SD and those outside this range.

## Results

### Demographics

Two-hundred and twenty patients were enrolled in this study. Their demographic details are listed in Table 1. The concomitant diagnoses in the study participants were as follows: systemic hypertension (n=130), diabetes mellitus (n=98), atrial fibrillation (n=85), ischemic heart disease (n=55), thyroid disorders (n=24), congestive cardiac failure (n=9) and bronchial asthma (n=15).

### Pharmacokinetic parameters

**Figure 1.**
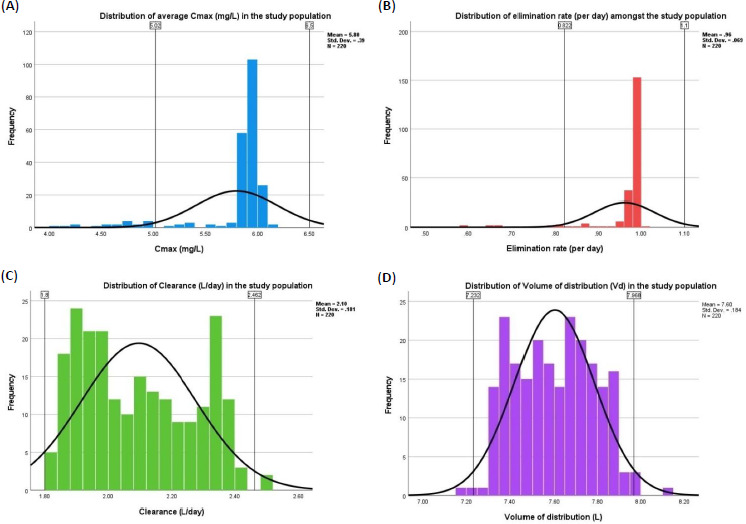
Distribution of pharmacokinetic parameters in the study population. (**A**) Distribution of *C*_max_ (mg/L); (**B**) elimination rate (per day); (**C**) clearance (L/day); (**D**) volume of distribution (L) in the study population

Mean (SD) *C*_max_ (mg/L) amongst the study participants was 5.8 (0.4); elimination rate (L/day) was 1 (0.1); CL (L/day) was 2.1 (0.2); and *V*_d_ (L) was 7.6 (0.2). Distributions of the pharmacokinetic parameters are depicted in Figure 1. Nineteen patients had their *C*_max_ outside the mean+1.96 SD and 14 had the same for elimination rate. Such patients were observed with significantly lower WSI and greater WCM (Table 2). For CL, two had their values lower than mean–1.96 SD; and for *V*_d_, two had lower than mean–2 SD, and four more than mean+2 SD. Due to the number constraints, we could not analyze the differences in any of the variables for CL and *V*_d_ of warfarin.

**Figure 2.**
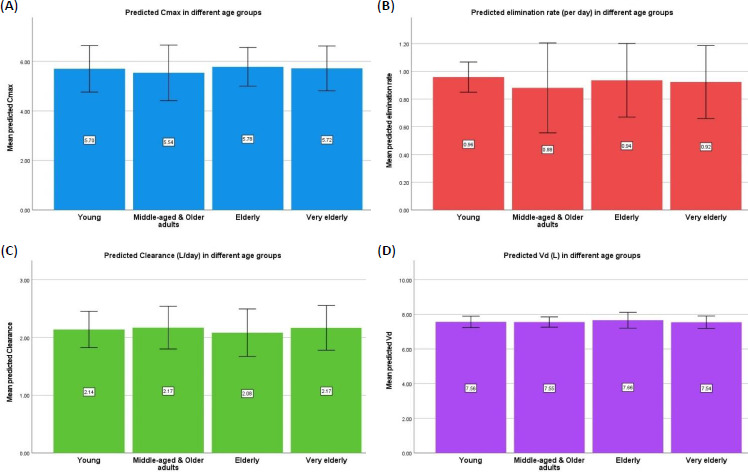
Predicted pharmacokinetic parameters of warfarin in different age groups. (**A**) Predicted *C*_max_ (mg/L); (**B**) predicted elimination (L/day); (**C**) predicted clearance (L/day); (**D**) predicted *V*_d_ (L) in different age groups.

**Figure 3.**
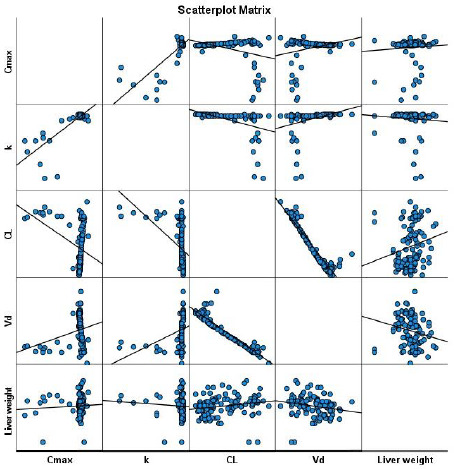
Scatter plot matrix between the pharmacokinetic parameters and the liver weight. Warfarin clearance and volume of distribution were significantly correlated with the weight of the liver. CL- clearance; *k* – elimination rate; *V*_d_ – volume of distribution

No significant differences were observed between the predicted pharmacokinetic parameters across the age groups (Figure 2). Similar values were obtained for males [*C*_max_ (mg/L – 5.8 (0.4); *k* (per day) – 1 (0.1); CL (L/day) – 2.1 (0.2); and *V*_d_ (L) – 7.6 (0.2)] and females [*C*_max_ (mg/L – 5.8 (0.4); *k* (per day) – 1 (0.05); CL (L/day) – 2.1 (0.2); and *V*_d_ (L) – 7.7 (0.2)]. Significant correlations were observed between *C*_max_ and CL (r = -0.4; p=0.0001), *V*_d_ (r = 0.28; p = 0.004) and *k* (r = 0.8; p = 0.0001) (Figure 3).

### Association between liver weights and predicted pharmacokinetic parameters

Scatterplot revealed significant correlations between warfarin CL (r=0.2; p=0.04) and *V*_d_ (r= -0.2; p=0.03) with weight of the liver (Figure 3).

## Discussion

We evaluated the pharmacokinetic parameters of warfarin using validated PK-PD model in our population. Two-hundred and twenty patients were recruited. The following were the predicted pharmacokinetic parameters: *C*_max_ (mg/L) was 5.8 (0.4); elimination rate (L/day) was 1 (0.1); CL (L/day) was 2.1 (0.2); and *V*_d_ (L) was 7.6 (0.2). Patients with *C*_max_ and elimination rate outside the mean+1.96 SD had significantly lower WSI and higher WCM. Significant correlations were observed between *C*_max_ with CL, *V*_d_, and *k* of warfarin. Significant correlations were also observed between CL and *V*_d_ of warfarin with liver weight of the study participants.

The pharmacokinetic parameters of racemic warfarin observed in the present study is like other populations [14]. Although it would be better to measure *S-* and *R- warfarin* concentrations, the clinical relevance of estimating enantiomers is limited. Ours is the first study exploring the relationship between pharmacokinetic parameters and WSI and WCM (the pharmacodynamic parameters). Lower WSI is corroborated with warfarin resistance. Genotyping of *vitamin K epoxide reductase complex* in our population might provide a clue towards the prevalence and depth of warfarin resistance. The present study is also the first in exploring the relationship between liver weight and pharmacokinetic parameters of warfarin. Though, liver weight has been argued to be a better corroborator of pharmacokinetic parameters in children, [15] its relationship in adults has not been studied.

The study is limited in not validating the pharmacokinetic parameters using real-time measurements of plasma warfarin concentrations. To conclude, we predicted pharmacokinetic parameters of warfarin from the validated pharmacokinetic-pharmacodynamic model in our population. More studies are needed exploring the relationship between various pharmacodynamic indices of warfarin and pharmacokinetic parameters of warfarin.

## Figures and Tables

**Table 1.** Demographic details of the study participants (N=220)

Variables	Values
Age (years)^$^	66.6 (13.4)
Male : Female	1 : 1
Body weight (kg)^$^	75.2 (18.1)
Predicted liver weight (g) ^$^	1148(265)
CHA_2_DS_2_-VASc score^$^	3.7 (1.5)
HASBLED score^$^	2.3 (1.1)
SAMe-TT_2_R_2_ score^$^	1.4 (0.6)
Duration of warfarin (days) ^$^	1027 (572.2)
Warfarin sensitive index ^$^	0.7 (0.4)
Log INR variability ^$^	-0.84 (0.2)
Warfarin composite measure score ^$^	-1.3 (1.2)
Number of patients with drugs with potential interaction [n (%)]	136 (61.8)

$ - Represented in mean (SD)

**Table 2.** Comparison of characteristics between those within and outside 95% of normal distribution curves in the study population

Variables	*C*_max_ (mg/L)			Elimination rate (per day)		
	Within 95% of ND (n= 201)	Outside 95% of ND (n= 19)	P-values	Within 95% of ND (n=206)	Outside 95% of ND (n=14)	P-values
Age (years) ^$^	67.1 (13.3)	61.7 (13.7)	0.7	68.6 (12.7)	64.2 (14.5)	0.6
Body weight (kg) ^$^	75 (18.4)	77.6 (14.1)	0.4	72.8(19)	81.6(12)	0.2
TTR (%) ^$^	65.6 (17.2)	64.7 (16.4)	0.6	65.7 (16.1)	65(21)	1
WSI ^$^	0.7 (0.4)	0.3 (0.1)	0.0001*	0.8 (0.4)	0.3 (0.1)	0.0001*
Log INR variability ^$^	-0.8 (0.2)	-0.9 (0.2)	0.06	-0.8 (0.2)	-0.9 (0.2)	0.06
WCM ^$^	-1.2 (1.2)	-1.8 (0.9)	0.004*	-1.1 (1.2)	-1.7 (1.1)	0.08
Potentially interacting drugs [n (%)]	123 (61.2)	12 (63.2)	0.9	135 (65.5)	8 (57.1)	0.5

Y-Young; M-Middle-aged & older adults; E – Elderly; VE – Very elderly; TTR – Time spent in therapeutic range; WSI – warfarin sensitive index; WCM – Warfarin composite measure; ND – Normal distribution

$ - Represented in mean (SD)

* - Statistically significant.
